# The Effects of Hedgehog on the RNA-Binding Protein Msi1 in the Proliferation and Apoptosis of Mesenchymal Stem Cells

**DOI:** 10.1371/journal.pone.0056496

**Published:** 2013-02-13

**Authors:** In-Sun Hong, Kyung-Sun Kang

**Affiliations:** 1 Adult Stem Cell Research Center, Seoul National University, Seoul, Republic of Korea; 2 Laboratory of Stem Cell and Tumor Biology, Department of Veterinary Public Health, Seoul National University, Seoul, Republic of Korea; The University of Adelaide, Australia

## Abstract

Human umbilical cord blood (UCB)-derived mesenchymal stem cells (MSCs) are essential tools for regenerative medicine due to their capacity for self-renewal and multi-lineage differentiation. As MSCs are found in very small numbers in various tissues, in vitro cell expansion is an essential step that is needed before these cells can be used in clinical applications. Therefore, it is important to identify and characterize factors that are involved in MSC proliferation and apoptosis. In the present study, we focused on Hedgehog (Hh) signaling because several studies have proposed that Hh signaling plays a critical role in controlling the proliferation of stem and progenitor cells. However, the molecular mechanisms underlying the effects on the proliferation and apoptosis of MSCs remain unclear. In this study, we evaluated the direct effects of Hh signaling on the proliferation and apoptosis of hUCB-MSCs as well as investigated potential downstream regulatory mechanisms that may be responsible for Hh signaling. We observed that the Hedgehog agonist purmorphamine enhanced cell proliferation and suppressed apoptosis through the RNA-binding protein Msi1 by regulating the expression of an oncoprotein (i.e., c-Myc), a cell cycle regulatory molecule (i.e., p21^CIP1,WAF1^ ) and two microRNAs (i.e., miRNA-148a and miRNA-148b). This study provides novel insights into the molecular mechanisms regulating the self-renewal capability of MSCs with relevance to clinical applications.

## Introduction

Mesenchymal stem cells (MSCs) are essential tools for regenerative medicine because of their proven potential to differentiate into multiple cell types. MSCs are derived from a variety of tissues, such as bone marrow and adipose tissue, and recent studies revealed the presence of these cells in umbilical cord blood (UCB) [1], 2. Isolating MSCs from UCB provides advantages, such as an easy ability to harvest cells with a high proliferation rate and high potential for differentiation into multiple tissue types [3]–[5].

In addition to multi-potency, the self-renewal capacity of MSCs is a crucial feature for their use in clinical applications of regenerative medicine. This capacity enables MSCs to retain the ability to differentiate into multiple tissue types throughout the entire lifespan of an individual organism [6]. As the clinical application of MSCs requires their extensive expansion in vitro, it is important to identify and characterize factors that are involved in their proliferation and apoptosis. However, it is still unclear how the self-renewal capacity of MSCs can be maintained in vitro.

Although a few signaling pathways have been implicated in the regulation of human MSC self-renewal capacity, these pathways have been confined to the effects of FGF [7], Activin A [8] and Wnt [9]. In this study, we were particularly interested in Hedgehog (Hh) signaling and the role it plays in the regulation of the self-renewal capacity of MSCs. Hh signaling is initiated by the binding of Hh to the transporter-like receptor Patched. Upon binding, Patched relieves its inhibition on Smoothened (Smo), which is a seven-pass transmembrane protein that transduces Hh signaling and, in turn, activates the transcription of Hh target genes in cells [10]. While it has been proposed that Hh signaling plays a critical role in controlling the proliferation [11] and differentiation [12] of stem and progenitor cells, the involvement of Hh signaling in the proliferation and apoptosis of MSCs is not clear, even though it is critical for the growth of many types of human cancers [13], [14]. Moreover, the molecular mechanisms underlying the effects of Hh signaling on the proliferation and apoptosis of MSCs remains unclear. Thus, the aims of our current study were twofold: 1) to evaluate the direct effects of Hh signaling on the proliferation and apoptosis of hUCB-MSCs and 2) to investigate novel downstream regulatory mechanisms that are responsible for the potential role of Hh signaling in proliferation and apoptosis.

Musashi (Msi) is an RNA-binding protein that is evolutionarily conserved across species, including xenopus, mouse, and human [15]. Two members of this family, Msi1 and Msi2, have been identified in mammals [16], [17]. Msi acts as a translational suppressor by binding to specific sites of mRNA targets. In mammals, Msi1 was originally found in neural stem/progenitor cells (NS/PCs) [18], and it was determined that Msi1 functions to maintain the self-renewal capability of NS/PCs [15], [19], [20]. Recently, the Msi1 protein was detected in non-CNS tissues and organs, including the eye [21], mammary gland [22], intestine [23], stomach [24], and hair follicle [25]. However, there is currently no information available on its role in the proliferation and apoptosis of MSCs. Therefore, the other objective of this study was to evaluate whether Msi1 can affect the proliferation and apoptosis of hUCB-MSCs as a novel downstream regulator of Hh signaling.

In the present study, we further investigate the potential downstream targets of Msi1, specifically p21^CIP1,WAF1^, c-Myc and various miRNAs, and their roles in the proliferation and apoptosis of MSCs. The cell cycle is negatively regulated by p21^CIP1,WAF1^, which inhibits cell proliferation by causing cell cycle arrest [26]. Recent studies suggest that the transient inhibition of p21^CIP1,WAF1^ results in a significant acceleration of MSC proliferation [27]. c-Myc is a well-known nuclear oncoprotein that exhibits multiple functions in cell proliferation, apoptosis and cellular transformation [28]. We recently reported that cell proliferation was dramatically decreased in hUCB-MSCs that were knoced down for c-Myc [29]. MicroRNAs are post-transcriptional regulators that bind to 3'-untranslated regions of target mRNA sequences, which usually results in target degradation or gene silencing [30]. It has been suggested that miRNAs play a regulatory role in the cell cycle of ES cells, adult stem cells and cancer stem cells [31]. However, no information is currently available on whether the expression of p21^CIP1,WAF1^, c-Myc or miRNAs is positively or negatively regulated by Msi1 during the proliferation and apoptosis of MSCs.

Taken together, the present study provides evidence for the regulation of hUCB-MSC proliferation and apoptosis by Hh signaling and suggests a novel mechanism for the controlled, in vitro expansion of MSCs for clinical applications in regenerative medicine.

## Materials and Methods

### The isolation and culture of hUCB–MSCs

Human umbilical cord blood-derived MSCs (hUCB-MSCs) were obtained from umbilical veins immediately after delivery with written consent from the mother and approval by the Boramae Hospital Institutional Review Board (IRB). UCB samples were mixed with HetaSep solution (Stem Cell Technology, Vancouver, BC, Canada) at a ratio of 5∶1. hUCB-MSCs were isolated by centrifugation for 20 min at 1200× g from a single-density Percoll layer. The cells were washed twice in PBS. Isolated cells were then cultured in growth medium consisting of D-media (Formula No. 78-5470EF, Gibco BRL) supplemented with EGM-2 SingleQuot and 10% FBS (Gibco BRL) at 37°C in a humidified atmosphere of 5% CO_2_ in air. After 3 days, non-adherent cells were removed by washing with PBS. All procedures for the preparation and utilization of hUCB-MSCs (IRB No. 0603/001-002) for research purposes were approved by the institutional review board of Seoul National University.

### Reagents

The Hedgehog agonist purmorphamine was purchased from Calbiochem (San Diego, CA). The siRNA for Musashi1 (Msi1) was purchased from Dharmacon, Inc. (Lafayette, CO). The anti-Hedgehog antibody was purchased from Millipore (Billerica, MA). The anti-Msi1 antibody was purchased from Cell Signaling Technologies (Beverly, MA). The anti-p21^CIP1,WAF1^ antibody was purchased from Millipore (Billerica, MA). The anti-c-Myc antibody was purchased from Calbiochem (San Diego, CA).

### Cell proliferation and viability

Cell proliferation and viability were evaluated by direct cell counting. In each experiment, the cells were plated at a 2×10^5^ seeding density and cultured with or without purmorphamine for 6 days. The cells were then enzymatically detached from the wells using 0.25% trypsin (Gibco). Viable and nonviable cells were detected by trypan blue (Sigma) and directly counted using a hemocytometer (Hausser Scientific Co., Horsham, PA).

### TUNEL assay

DNA strand breaks in apoptotic cells were measured with a TUNEL assay using the In-situ Detection Kit (Roche Molecular Biochemicals, Germany). The samples were fixed with 4% paraformaldehyde in PBS for 15 min and incubated in a 0.1% ice-cold Triton X-100 solution for permeabilization for 10 min according to the manufacturer's instructions. The cells were then washed 3 times with PBS and incubated with 50 µl of TUNEL reaction mixture at 37°C for 60 min in a dark, humidified chamber. The cells were then rinsed three times in PBS. The results were visualized by fluorescent microscopy.

### Western blot analysis

After the aforementioned treatments, cells were washed twice with ice-cold PBS and lysed in ice-cold RIPA buffer containing phosphatase inhibitor cocktail I (Sigma Chemical, St. Louis, MO). The protein samples were then separated by SDS-PAGE and transferred onto nitrocellulose membranes. The membranes were blocked in PBS containing 5% skim milk at room temperature for 1 h and then washed in PBS and incubated overnight at 4°C with the appropriate primary antibodies for protein detection. Secondary antibodies were diluted to a 1∶3000 concentration in 10 ml PBS containing 5% skim milk. Each membrane was incubated with the appropriate, diluted secondary antibodies for 2 h at 37°C and then rinsed three times with PBS containing 0.05% Tween 20. Each band detected by western blot was quantified with the Scion Image Software (Scion, Frederick, MD) using β-actin as an internal control.

### Real-time PCR

RNA was extracted from the cells using 1 ml of Trizol reagent (Invitrogen, Life Technologies) according to the manufacturer's instructions. Total RNA was reverse-transcribed into cDNA using a first-strand cDNA synthesis kit (GE Healthcare Biosciences, Piscataway, NJ). The primers used for SYBR Green real-time RT-PCR were designed using Primer Express Software v2.0 (Applied Biosystems, Foster City, CA).

### Msi1 siRNA transfection

Shortly before transfection, 2×10^6^ cells were seeded into 6-well dishes and incubated at 37°C. ON-TARGET plus the Msi1 siRNA or a control siRNA were added to the cell medium (100 µl of DMEM medium containing FBS without antibiotics) at a final concentration of 50 nM; the cells were then incubated for 15 min at room temperature with an RNA transfection reagent (Lipofectamine RNAiMAX, Invitrogen, Carlsbad, CA, USA). Then, the cells were incubated with the siRNA transfection solution for 48 h. After 48 h, the transfection medium was replaced by culture medium consisting of D-media supplemented with EGM-2 and 10% FBS.

### Statistical analysis

Each set of experiments was performed in triplicate. The results for each experiment were presented as the mean ± SD. The data were assessed using one-way analysis of variance (ANOVA), and significant results were further analyzed using Tukey's multiple comparison test. Statistical significance was defined at a *P* level of *<0.05*.

## Results

### MSC-specific effects of Hedgehog signaling on the proliferation of hUCB-MSCs

**Figure 1:**
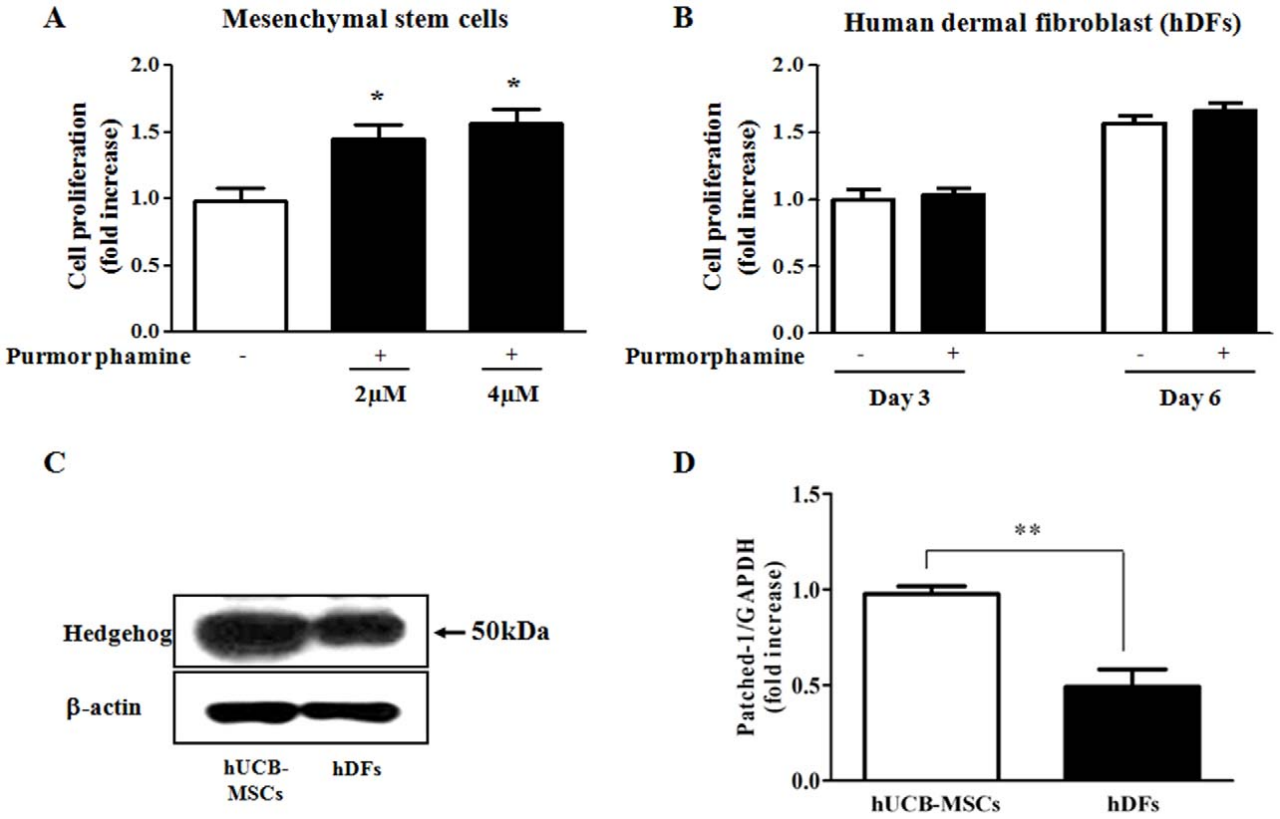
The Hedgehog agonist purmorphamine specifically promotes the proliferation of MSCs. hUCB-MSCs and hDFs were incubated in standard culture medium with or without purmorphamine (4 µM). Cell numbers were then obtained by direct cell counting with a hemocytometer. (**A**) Purmorphamine exposure significantly increased MSC proliferation in a dose-dependent manner compared to negative controls. (**B**) Purmorphamine treatment did not affect cell proliferation in hDFs compared to negative controls. (**C, D**) After preculture for 24 h, protein and mRNA were isolated from human dermal fibroblasts (hDFs) and hUCB-MSCs. The level of Hh expression was assessed by western blotting, and Patched-1 mRNA levels were assessed by real-time PCR. The relative expression levels of Hh and Patched-1 in hUCB-MSCs were higher than the levels observed in hDFs. β-actin was used as an internal control. The results are shown as the mean ± SD from three independent experiments. * P<0.05, ** P<0.01, and *** P<0.001.

We examined the response of hUCB-MSCs to Hedgehog stimulation. Cell proliferation levels were evaluated by direct cell counting after purmorphamine treatment. Purmorphamine exposure significantly increased cell proliferation in a dose-dependent manner compared with the negative controls (Fig. 1A). To determine whether this effect was stem cell-specific, we tested whether Hh signaling affected the proliferation of human dermal fibroblasts (hDFs) when compared with MSCs by direct cell counting after purmorphamine treatment. Purmorphamine treatment did not affect cell proliferation in hDFs compared with the negative controls (Fig. 1B). Taken together, these results indicate that the activation of Hh signaling specifically enhances the proliferation of hUCB-MSCs and not hDFs.

Hh signaling is initiated by the binding of Hh to the transporter-like receptor Patched. It is therefore reasonable to assume that differential expression levels of Hedgehog and its receptor in hUCB-MSCs and hDFs likely contribute to the MSC-specific responses to Hh signaling. We examined whether the expression levels of Hh and its receptor, Patched-1, are lower in human dermal fibroblasts (hDFs) compared with hUCB-MSCs by western blot analysis and real-time PCR. The relative expression levels of Hh and Patched-1 in hUCB-MSCs were higher than the levels observed in hDFs (Figs. 1C, D).

### The activation of Hh signaling enhances Msi1 protein expression and highly correlates with purmorphamine's anti-apoptotic effects

**Figure 2:**
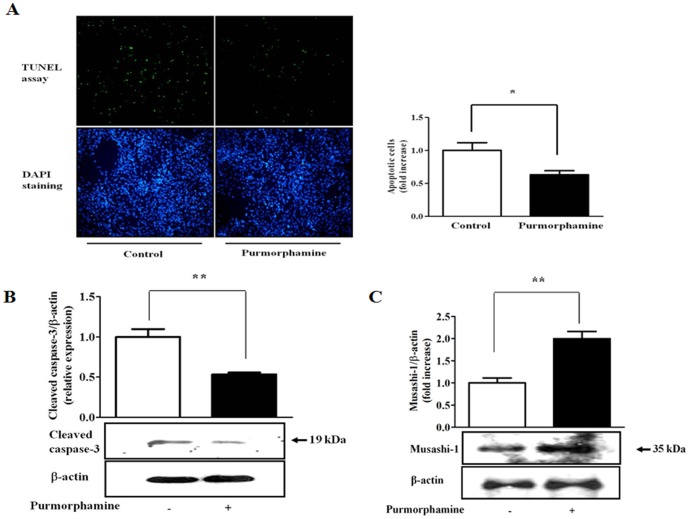
Hedgehog stimulation suppresses apoptosis through the activation of Msi1. hUCB-MSCs were cultured in serum-free medium and then treated with or without purmorphamine (4 µM). Apoptotic levels were assessed with a TUNEL assay and western blotting. Apoptotic cells were quantified by counting the number of TUNEL-positive cells. Purmorphamine treatment for 72 h significantly decreased the number of TUNEL-positive cells (**A**) and the expression level of cleaved caspase-3 (**B**). The blue staining (DAPI) identifies cell nuclei. (**C**) The expression level of Msi1 was assessed by western blot. 4 µM purmorphamine treatment for 72 h significantly increased Msi1 expression. β-actin was used as an internal control. The results are shown as the mean ± SD from three independent experiments. * P<0.05, ** P<0.01, and *** P<0.001.

We subsequently investigated whether the effect of purmorphamine on the proliferation of hUCB-MSCs is correlated with anti-apoptotic effects. We performed TUNEL assays to identify apoptotic cells, which are characterized by the inclusion of densely stained circular bodies that represent fragmented DNAs resulting from apoptosis. To further confirm the anti-apoptotic effects of purmorphamine, western blot analyses were used to evaluate the expression levels of activated caspase-3. hUCB-MSCs were cultured in serum-free medium and treated with or without purmorphamine (4 µM). Purmorphamine treatment for 72 h significantly decreased both the number of TUNEL-positive cells (Fig. 2A) and the expression level of activated caspase-3 (Fig. 2B) compared with the controls.

To determine the mechanisms underlying the anti-apoptotic and pro-proliferative effects of Hh signaling on hUCB-MSCs, we first examined whether Hh signaling affected the expression of Msi1, which is responsible for the self-renewal capability of many types of stem cells. As illustrated in Fig. 2C, we found that Msi1 expression was significantly increased after purmorphamine stimulation. This result suggests that Msi1 is involved in Hh signaling as a downstream target during cell proliferation.

### The effects of Msi1 knockdown on cell proliferation and apoptosis

**Figure 3:**
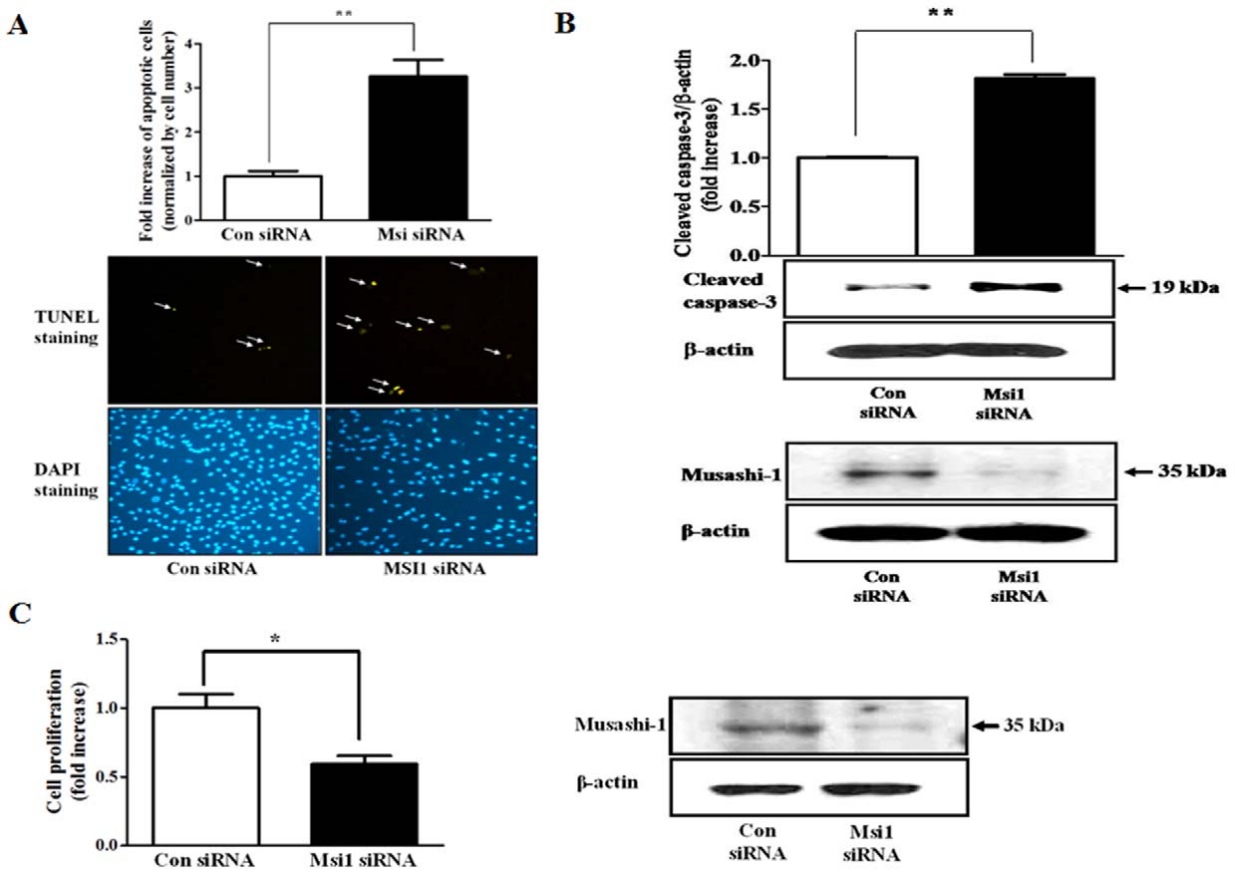
The effects of Msi1 knockdown on cell proliferation and apoptosis. After preculture for 24 h, hUCB-MSCs were transfected with 50 nM Msi1 siRNA for 48 h. The transfection of 50 nM Msi1 siRNA efficiently reduced Msi1 protein levels. The transfection of 50 nM Msi1 siRNA also significantly increased the number of TUNEL-positive cells (**A**), as well as the expression level of activated caspase-3 (**B**), compared with the controls. (**C**) The transfection of 50 nM Msi1 siRNA significantly increased cell proliferation compared to controls. The blue staining (DAPI) identifies cell nuclei. β-actin was used as an internal control. The results are shown as the mean ± SD from three independent experiments. * P<0.05, ** P<0.01, and *** P<0.001.

To further elucidate the functional roles of Msi1 in the proliferation and apoptosis of MSCs, hUCB-MSCs were first transfected with Msi1 siRNA, and then, TUNEL assays and direct cell counting were performed. We found that the transfection of 50 nM Msi1 siRNA significantly increased the number of TUNEL-positive cells (Fig. 3A), as well as the expression level of activated caspase-3 (Fig. 3B), compared with the controls. We also found that the knockdown of Msi1 significantly decreased cell number compared with the negative controls by increasing apoptosis (Fig. 3C).

### Msi1 functions as a downstream regulator of Hh signaling

**Figure 4:**
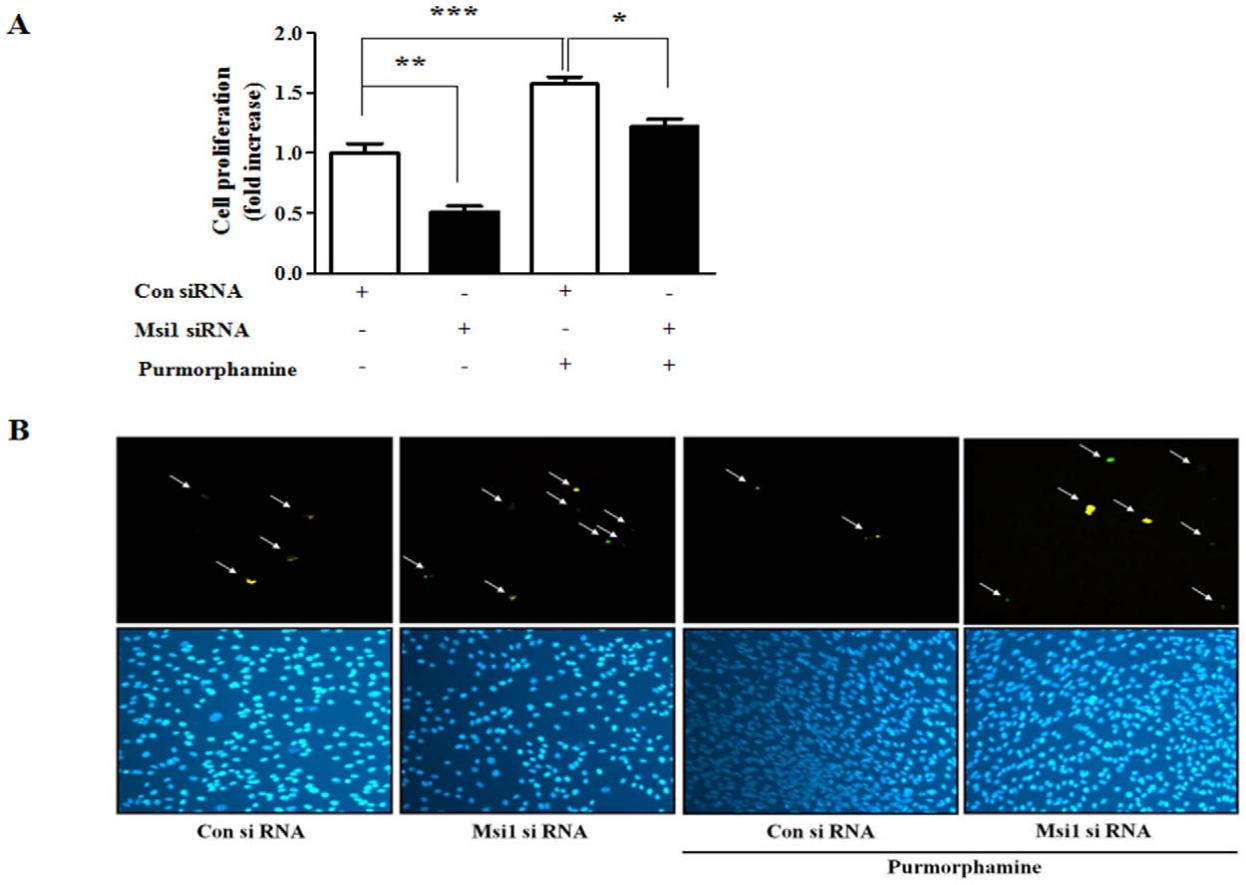
The attenuating effects of Msi1 knockdown on Hh signaling in MSC proliferation and apoptosis. Cells were treated with or without 4 µM purmorphamine and transfected with or without 50 nM Msi1 siRNA. The cell proliferation levels were then evaluated by direct cell counting, and apoptosis levels were assessed with a TUNEL assay and western blotting. Purmorphamine increased cell proliferation (**A**) and decreased TUNEL-positive cell numbers (**B**), while the 50 nM Msi1 siRNA transfection produced the opposite effects. The knockdown of Msi1 attenuated the above effects of purmorphamine treatment. The blue staining (DAPI) identifies cell nuclei. The results are shown as the mean ± SD from three independent experiments. * P<0.05, ** P<0.01, and *** P<0.001.

To further elucidate the role of Msi1 as a downstream target of Hh signaling during the proliferation and apoptosis of MSCs, hUCB-MSCs were transfected with Msi1 siRNA and then treated with 4 µM purmorphamine. As expected, the knockdown of Msi1 decreased cell proliferation, and purmorphamine increased cell proliferation. In contrast, the stimulatory effects of purmorphamine on cell proliferation were significantly attenuated by Msi1 knockdown (Fig. 4A). We next evaluated the effects of Msi1 knockdown on Hh stimulation during apoptosis. The knockdown of Msi1 increased the number of TUNEL-positive cells, while purmorphamine decreased the number of TUNEL-positive cells. Furthermore, the inhibitory effects of purmorphamine on apoptosis were attenuated by the knockdown of Msi1 (Fig. 4B).

### The effects of purmorphamine on p21^CIP1,WAF1^ and c-Myc are mediated through Msi1

**Figure 5:**
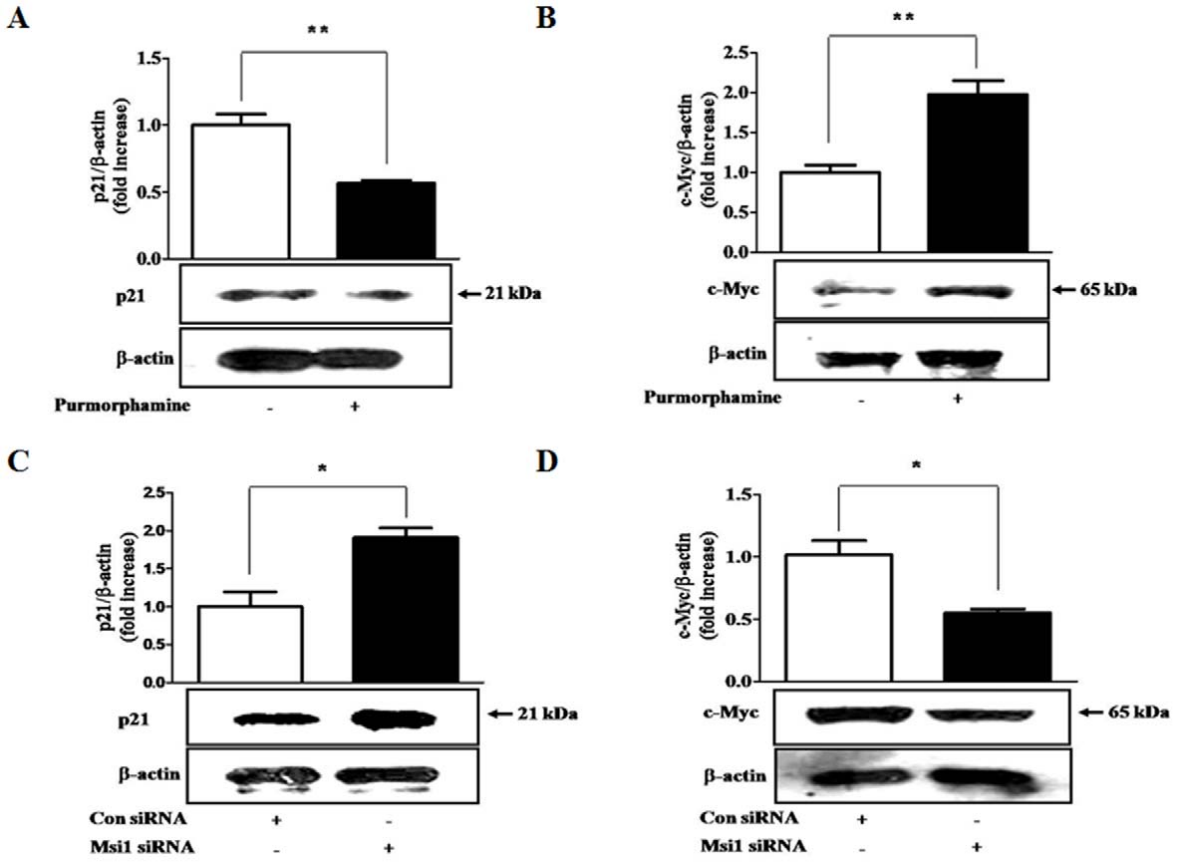
The effects of purmorphamine and Msi1 knockdown on p21^CIP1,WAF1^ and c-Myc expression. hUCB-MSCs and hDFs were incubated in standard culture medium with or without 4 µM purmorphamine for 3 days. p21^CIP1,WAF1^ and c-Myc expressions levels were then assessed by western blotting. Purmorphamine treatment decreased and increased the expression of p21^CIP1,WAF1^ (**A**) and c-Myc (**B**), respectively. After preculture for 24 h, hUCB-MSCs were transfected with 50 nM Msi1 siRNA for 48 h. The transfection of 50 nM Msi1 siRNA significantly increased and decreased the expression levels of p21^CIP1,WAF1^ (**C**) and c-Myc (**D**), respectively. β-actin was used as an internal control. The results are shown as the mean ± SD from three independent experiments. * P<0.05, ** P<0.01, and *** P<0.001.

**Figure 6:**
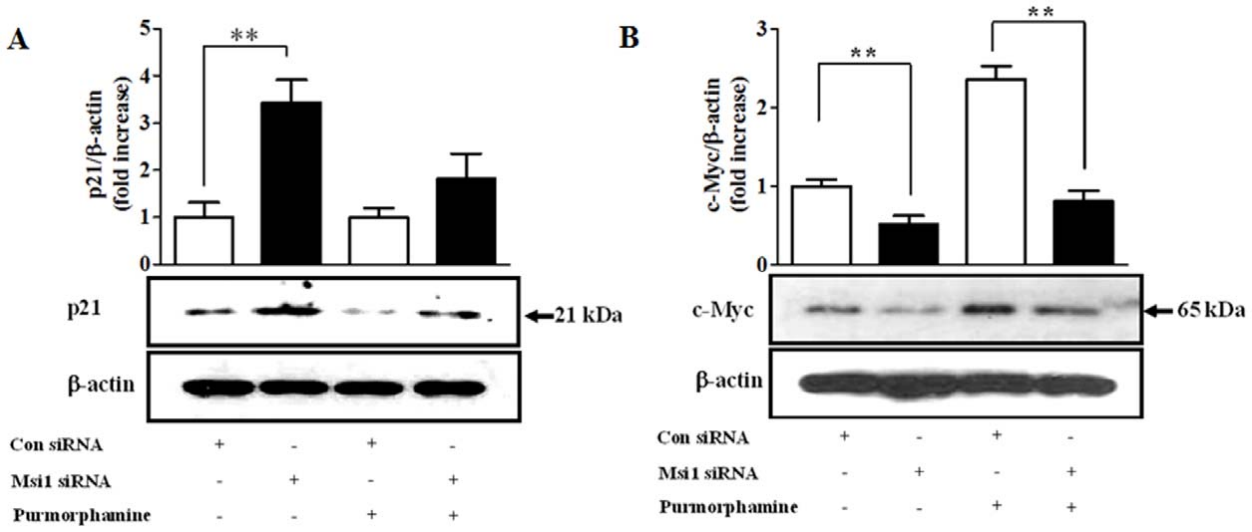
The effects of purmorphamine on p21^CIP1,WAF1^ and c-Myc are mediated through Msi1. hUCB-MSCs were first transfected with 50 nM Msi1 siRNA for 48 h and then treated with or without purmorphamine (4 µM) for 3 days. The transfection of 50 nM Msi1 siRNA reversed the inhibitory (**A**) and stimulatory (**B**) effects of purmorphamine on p21^CIP1,WAF1^ and c-Myc expression, respectively. β-actin was used as an internal control. The results are shown as the mean ± SD from three independent experiments. * P<0.05, ** P<0.01, and *** P<0.001.

We evaluated the direct effects of Hh signaling on the expression of p21^CIP1,WAF1^ and c-Myc and determined whether Msi1 was involved in these interactions. As illustrated in Figs. 5A and B, purmorphamine treatment decreased and increased the expression of p21^CIP1,WAF1^ and c-Myc, respectively. We also found that the knockdown of Msi1 increased and decreased the expression levels of p21^CIP1,WAF1^ and c-Myc, respectively (Figs. 5C, D). To further determine whether Msi1 is directly involved in these interactions, we examined the expression of p21^CIP1,WAF1^ and c-Myc following Msi1 siRNA transfection. The knockdown of Msi1 reversed the inhibitory (Fig. 6A) and stimulatory (Fig. 6B) effects of purmorphamine on p21^CIP1,WAF1^ and c-Myc expression, respectively.

### The regulatory roles of miR-148a and miR-148b as downstream targets of Msi1

**Figure 7:**
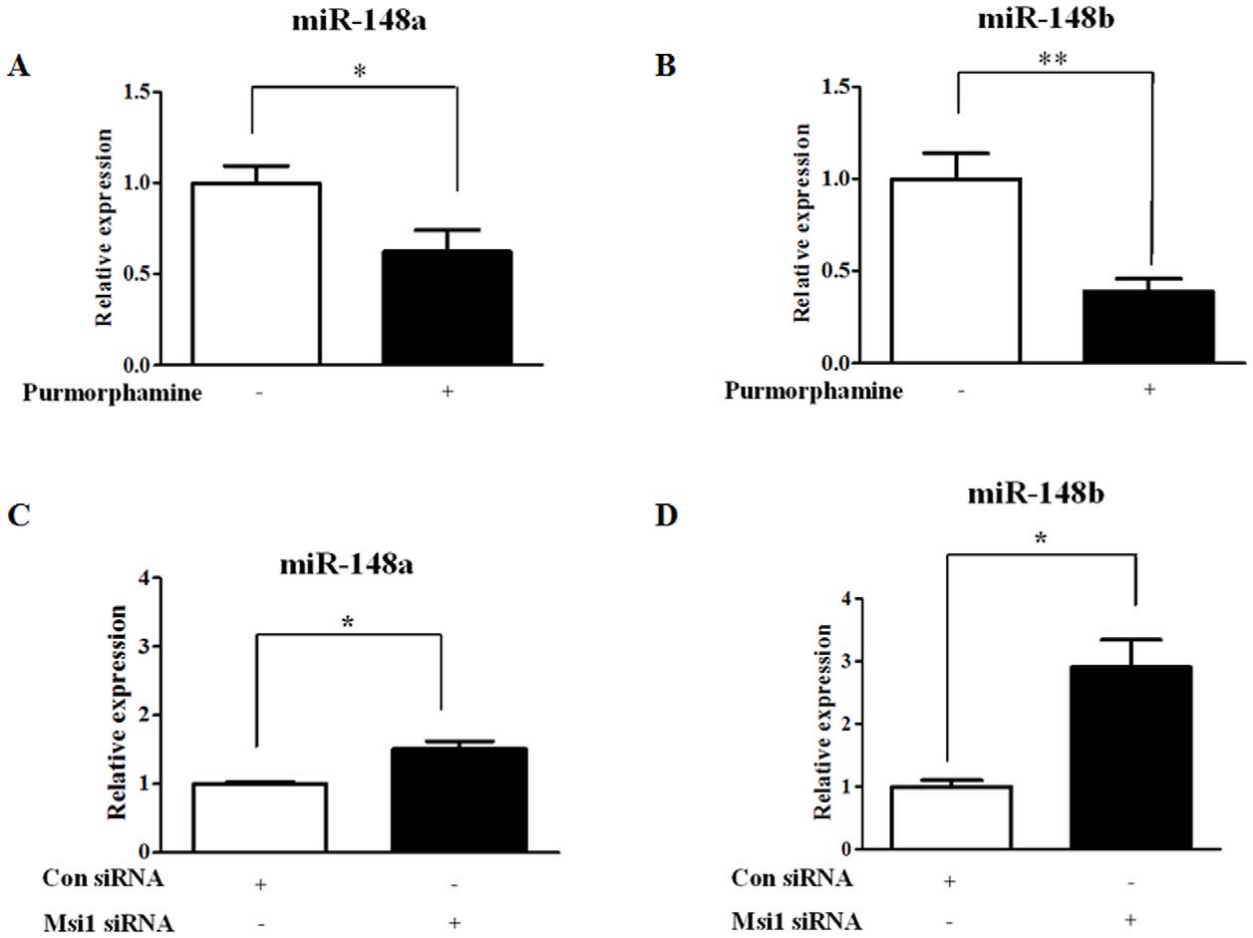
The effects of purmorphamine and Msi1 knockdown on miR-148a and miR-148b expression. hUCB-MSCs were treated with 4 µM purmorphamine for 3 days or transfected with 50 nM Msi1 siRNA, and then, the expression profiles of the miRNAs were analyzed by real-time PCR. The expression levels of miR-148a (**A**) and miR-148b (**B**) were decreased by purmorphamine treatment. The transfection of 50 nM Msi1 siRNA increased the expression levels of miR-148a (**C**) and miR-148b (**D**). The results are shown as the mean ± SD from three independent experiments. * P<0.05, ** P<0.01, and *** P<0.001.

**Figure 8:**
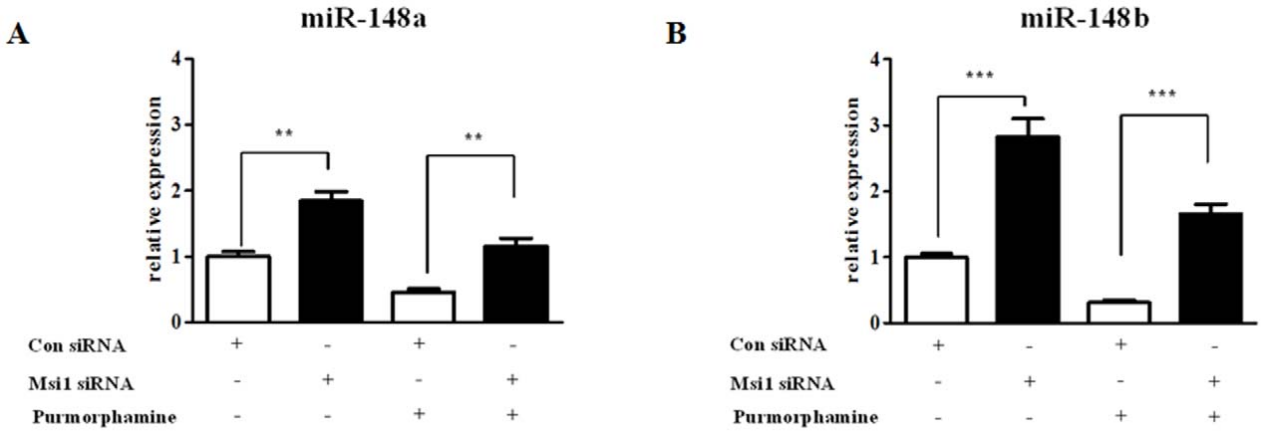
The effects of purmorphamine on miR-148a and miR-148b are mediated through Msi1. hUCB-MSCs were first transfected with 50 nM Msi1 siRNA for 48 h and then treated with or without 4 µM purmorphamine for 3 days. The expression profiles of the miRNAs were then analyzed using real-time PCR. Purmorphamine treatment attenuated the stimulatory effects of Msi1 knockdown on miR-148a (**A**) and miR-148b (**B**) expression. The results are shown as the mean ± SD from three independent experiments. * P<0.05, ** P<0.01, and *** P<0.001.

We analyzed the expression levels of miR-148a and miR-148b with or without 4 µM purmorphamine treatment using real-time PCR analysis. As shown in Figs. 7A and B, the expression levels of miR-148a and miR-148b were decreased by purmorphamine treatment but it was more marked in miR-148b than miR-148a. Because Hh signaling exerts its functions on MSCs through Msi1, we first assessed whether Msi1 could regulate the expression of miR-148a and miR-148b. The knockdown of Msi1 significantly increased the expression levels of miR-148a and miR-148b (Figs. 7C, D). We further determined whether Msi1 is involved in the interactions between Hh signaling and miR-148a and miR-148b. In these experiments, we found that purmorphamine treatment attenuated the stimulatory effects of Msi1 knockdown on miR-148a and miR-148b expression (Figs. 8A, B).

## Discussion

Human mesenchymal stem cells (MSCs) may prove to be a valuable resource for regenerative medicine because of their long-term self-renewal capability and capacity to differentiate into multiple cell types. As the clinical application of MSCs requires extensive in vitro expansion, a detailed understanding of the molecular mechanisms underlying the proliferation and apoptosis of MSCs is of particular importance. However, it is still unclear how the self-renewal capacity of MSCs can be maintained in vitro.

It has been suggested that various growth factors, including Activin A [8], Wnt [9] and FGF [7], stimulate MSC proliferation via different signaling mechanisms; however, other regulatory factors also seem to be involved in the proliferation and apoptosis of MSCs. Sonic hedgehog (Shh) is a member of the Hedgehog (Hh) family of secreted signaling proteins and exhibits diverse functions during vertebrate development [32]. Recently, it has been proposed that aberrant Hh signaling plays a causal role in many types of human cancer stem cells, such as leukemia [33], medulloblastoma [34], and gastric cancer [35] stem cells. Additionally, accumulating evidence from several groups suggests that Hh signaling plays a critical role in controlling the proliferation of various types of stem cells, including neural [36], mammary [37], and hair follicle [38] stem cells. However, there is currently no information available on whether Hh signaling regulates the proliferation and apoptosis of human MSCs. In this study, we demonstrate for the first time that the Hedgehog agonist purmorphamine enhanced the proliferation capability of hUCB-MSCs. Furthermore, the activation of the Hh signaling pathway suppressed apoptosis by inhibiting caspase-3 activity.

There is increasing evidence and recognition of the functional roles of RNA-binding proteins in cell proliferation and/or apoptosis via the regulation of target oncoprotein and cell cycle regulatory molecule expression. Recently, Msi1 protein expression was identified in non-CNS tissues and organs [22], [24]. Msi1 is up-regulated in many cancers, such as medulloblastoma [39], hepatocellular carcinoma [40] and lung cancer [41]. However, no information is available on Msi1 expression and function in the proliferation and apoptosis of human MSCs. Another important finding of this study is that the knockdown of Msi1 significantly decreased cell proliferation and increased caspase 3-mediated apoptosis. Additionally, the effects of Hh signaling on the proliferation and apoptosis of hUCB-MSCs were attenuated by Msi1 knockdown, which suggests that Msi1 is required for Hh signaling to induce cell proliferation and inhibit apoptosis in hUCB-MSCs. Taken together, we show for the first time that Msi1 plays a novel role as an anti-apoptotic survival factor in hUCB-MSCs.

In the present study, we further investigated two novel downstream targets of Msi1 for their roles in the proliferation and apoptosis of MSCs, specifically an oncoprotein, c-Myc, and a cell cycle regulatory molecule, p21^CIP1,WAF1^ . c-Myc belongs to a family of basic helix-loop-helix leucine zipper (bHLHLZ) transcription factors. c-Myc levels are down-regulated during cellular senescence and rapidly increased by various growth factors, and the overexpression of c-Myc protein is a common event in a variety of tumors [42], [43]. Recent studies suggest that c-Myc is essential for the proliferation of various stem cells, including hematopoietic [44], mesenchymal [45] and epidermal [46] stem cells, but there is no information available on whether Msi1 enhances or suppresses c-Myc expression during the proliferation and apoptosis of MSCs. The knockdown of Msi1 reversed the stimulatory effects of purmorphamine on c-Myc expression levels. These results suggest that Msi1 is an upstream regulator of c-Myc, which mediates in the regulation of MSC proliferation and apoptosis by Hh signaling.

p21^CIP1,WAF1^ (also known as cyclin-dependent kinase inhibitor) is an important negative regulator of the cell cycle [47]. Recent research reported that the inhibition of p21^CIP1,WAF1^ results in the significant acceleration of mesenchymal [27], neural [48] and intestinal [49] stem cell proliferation. These studies suggested a functional role for p21 in controlling stem cell proliferation and apoptosis in various adult stem cells. However, the underlying mechanisms involved are unclear, and no information is currently available about the interactions between Msi1 and p21^CIP1,WAF1^. Knockdown of Msi1 did not completely abrogate the inhibitory and stimulatory effects of purmorphamine on p21^CIP1,WAF1^ and c-Myc expression, respectively. At present, it is uncertain if these partial effects are due to incomplete Msi1 knockdown and/or the regulatory effects of purmorphamine on p21CIP1,WAF1 and c-Myc expression are partially mediated through other signaling pathways. Further studies are needed to assess the effects of Msi1 overexpression on p21^CIP1,WAF1^ and c-Myc expression to corroborate our findings.

miRNAs are small non-coding RNAs that regulate gene expression through sequence-specific interactions with their target mRNAs. miRNAs are involved in various biological processes by affecting the expression of target genes [30]. Recent studies show that a number of miRNAs are involved in the self-renewal and pluripotency capacities of various stem cell types [50]–[52]. However, the identification of specific miRNAs and their potential regulatory effects on MSC proliferation and apoptosis remain unclear. In this study, we showed for the first time that the expression levels of miR-148a and miR-148b were decreased by purmorphamine treatment but it was more marked in miR-148b than miR-148a. However, the molecular mechanisms that might contribute to these differential responses to purmorphamine treatment remain largely unknown. Further studies are needed to assess factors for these differential responses

Furthermore, we have shown that knockdown of Msi1 increased the expression levels of miR-148a and miR-148b, and purmorphamine treatment partially attenuated the stimulatory effects of Msi1 knockdown on miR-148a and miR-148b expression. These results indicate that miR-148a and miR-148b may be involved in MSC proliferation and apoptosis as novel downstream regulators of Msi1. However, whether these partial effects are due to incomplete Msi1 knockdown and/or the regulatory effects of purmorphamine on p21^CIP1,WAF1^ and c-Myc expression are partially mediated through other signaling pathways remains unknown. Further studies will be needed to demonstrate these results to corroborate our findings.

**Figure 9:**
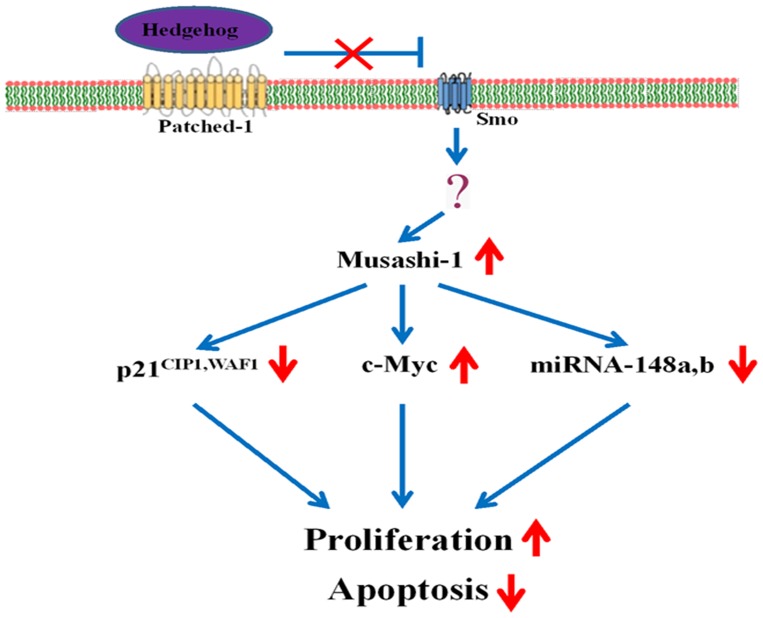
A schematic diagram showing the proposed Hedgehog signaling cascade that regulates the apoptosis and proliferation of MSCs. The Hedgehog agonist purmorphamine enhanced cell proliferation and suppressed apoptosis through the RNA-binding protein Msi1 by regulating the expression of an oncoprotein (i.e., c-Myc), a cell cycle regulatory molecule (i.e., p21^CIP1,WAF1^ ) and two miRNAs (i.e., miRNA-148a and miRNA-148b).

In conclusion, we demonstrated that the Hedgehog agonist purmorphamine enhanced cell proliferation and suppressed apoptosis through Msi1 by regulating the expression of an oncoprotein (c-Myc), a cell cycle regulatory molecule (p21^CIP1,WAF1^) and two miRNAs (miRNA-148a and miRNA-148b) (Fig. 9). However, the signaling pathway between Hh receptor and Msi1 remains largely unknown. Recent studies have shown that Notch and PI3K/Akt signaling pathways interact directly with Msi1 in glioblastoma, and that the interaction promotes cell growth [53]. This study provides novel insights into Hedgehog/Msi1 signaling and its role in the regulation of the self-renewal capability of hUCB-MSCs.
