# Correction: The Effects of Hedgehog on the RNA-Binding Protein Msi1 in the Proliferation and Apoptosis of Mesenchymal Stem Cells

**DOI:** 10.1371/annotation/da199b79-3c4a-4213-b28a-78f77abfa1fb

**Published:** 2013-04-16

**Authors:** In-Sun Hong, Kyung-Sun Kang

There were errors in the Funding statement. The correct statement is:

This work was supported by the Global Frontier Project grant (NRF-M1AXA002) and the the Bio & Medical Technology Development Program (No. 2010-0020265) of the National Research Foundation (NRF) funded by the Ministry of Education, Science and Technology of Korea, and the Ministry of Education, Science and Technology (MEST). The funders had no role in study design, data collection and analysis, decision to publish, or preparation of the manuscript. 

